# Efficacy of Mass Azithromycin Distribution for Reducing Childhood Mortality Across Geographic Regions

**DOI:** 10.4269/ajtmh.18-1003

**Published:** 2019-02-07

**Authors:** Travis C. Porco, Catherine E. Oldenburg, Ahmed M. Arzika, Khumbo Kalua, Zakayo Mrango, Catherine Cook, Elodie Lebas, Robin L. Bailey, Sheila K. West, Assaf P. Oron, Jeremy D. Keenan, Thomas M. Lietman, for the MORDOR Study Group

**Affiliations:** 1 Francis I. Proctor Foundation for Research in Ophthalmology, San Francisco, California;; 2 Department of Ophthalmology, University of California, San Francisco, San Francisco, California;; 3 Department of Epidemiology and Biostatistics, University of California, San Francisco, San Francisco, California;; 4 The Carter Center, Niamey, Niger;; 5 Blantyre Institute for Community Outreach, Blantyre, Malawi;; 6 College of Medicine, University of Malawi, Blantyre, Malawi;; 7 National Institute for Medical Research, Dar es Salaam, Tanzania;; 8 London School of Hygiene & Tropical Medicine, London, United Kingdom;; 9 The Dana Center, Johns Hopkins University School of Medicine, Baltimore, Maryland;; 10 Institute for Disease Modeling, Bellevue, Washington

## Abstract

Mass azithromycin distribution has been shown to reduce all-cause mortality in preschool children in sub-Saharan Africa. However, substantial heterogeneity in the apparent effect has been noted across geographic settings, suggesting a greater relative benefit in higher mortality settings. Here, we evaluated the relationship between the underlying mortality rate and the efficacy of azithromycin for the prevention of child mortality using data from multiple sites in Ethiopia, Malawi, Niger, and Tanzania. Between regions, we find no strong evidence of effect modification of the efficacy of azithromycin distribution for the prevention of child mortality by the underlying mortality rate (*P* = 0.12), although a modest effect is consistent with our findings. Higher mortality settings could be prioritized, however, because of the larger number of deaths which could be averted with azithromycin distribution.

## INTRODUCTION

Small trials of mass azithromycin distribution for trachoma control suggested that mass azithromycin may reduce postneonatal childhood mortality.^1–3^ In the original primary analyses, the Trachoma Amelioration in northern Amhara (TANA) study demonstrated an odds ratio of 0.51 for mortality in the azithromycin treatment group (95% CI: 0.29–0.90), for an overall reduction in child mortality in communities receiving azithromycin compared with delayed azithromycin. In the Niger component of the Partnership for the Rapid Elimination of Trachoma (PRET) study, biannual treatment of children led to a mortality rate ratio of 0.81 (95% CI: 0.66–1.0), an overall reduction in child mortality compared with annual treatment of the entire community. This finding was confirmed in the *Macrolides Oraux pour Réduire les Décès avec un Oeil sur la Résistance* (MORDOR) study, a larger three-country study that demonstrated a 13.5% (95% CI: 6.7–19.8%) reduction in mortality with biannual distributions^4^ in children aged 1–59 months.

Overtreatment with antibiotics has clear downsides. Distribution of azithromycin for trachoma control has been shown to select for macrolide resistance in some organisms.^5–8^ However, identifying areas which maximize the efficacy of azithromycin for prevention of child mortality could aid in limiting distributions to regions where it may be most effective, and thus limit antibiotic consumption. In both subgroup analyses of MORDOR and the pooled analysis,^9^ effects appeared to be larger in areas with higher mortality. However, as childhood mortality is a statistically rare event even in high-burden settings, these analyses were not designed to provide evidence of a statistically significant relationship between mortality rate and efficacy of azithromycin distribution. Here, we assess the dependence of the efficacy of mass azithromycin distributions on the underlying childhood mortality rate in three trials that have evaluated azithromycin for the prevention of child mortality.^1,2,4^

## METHODS

### Data.

#### Trachoma Amelioration in Northern Amhara.^1^

Communities in the Goncha Siso Enese *woreda* of Amhara, Ethiopia, were randomized to one of four arms: 1) annual azithromycin distribution to all individuals aged 1 year and older, 2) biannual azithromycin distribution to all individuals aged 1 year and older, 3) quarterly treatment of children aged 1–9 years, or 4) delayed treatment.

Communities were followed for 12 months. A door-to-door census was conducted in all communities, and all-cause mortality as per the census was a prespecified primary outcome. Children younger than 12 months were not treated in TANA, and thus were excluded from analysis. For this analysis, we included only children aged 1–5 years.

#### Partnership for the Rapid Elimination of Trachoma.^2^

Communities in Matameye, Niger, were randomized to one of four arms: 1) annual azithromycin distribution with an 80% coverage target, 2) annual azithromycin distribution with a 90% coverage target, 3) biannual azithromycin distribution to children aged 6 months to 12 years with an 80% coverage target, or 4) biannual azithromycin distribution to children with a 90% coverage target.^10–12^ Mortality was determined via a door-to-door census conducted before each treatment phase. Communities were followed for 36 months.

*Macrolides Oraux pour Réduire les Décès avec un Oeil sur la Résistance*^4^ communities in the districts of 1) Mangochi, Malawi; 2) Boboye and Loga, Niger; and 3) Kilosa and Gairo, Tanzania, were randomized to biannual mass azithromycin distribution to all children aged 1–59 months or biannual matching placebo. A house-to-house enumerative census was conducted in 6-month study periods. At each follow-up census, vital status (alive, dead, or unknown) was documented for children from previous censuses. The prespecified primary outcome was community-level all-cause mortality as determined by the census.

Each study was reviewed and approved by the applicable institutional review boards, including the Committee on Human Research at the University of California, San Francisco (TANA, PRET, MORDOR), the Ethiopian Science and Technology Commission (TANA), and the Institutional Review Boards at Emory University (TANA, MORDOR), the College of Medicine, University of Malawi, Blantyre (MORDOR), the Niger Ministry of Health (PRET, MORDOR), the Tanzanian National Institute for Medical Research (MORDOR), the London School of Hygiene and Tropical Medicine (MORDOR), and the Johns Hopkins University School of Medicine (MORDOR). Complete methods for each study have been previously reported.^1,2,4^

### Statistical analysis.

For the three MORDOR study sites, communities were aggregated into zones. We divided the range of longitude and latitude for each country into equal segments, resulting in an *r* × *c* grid which defines a zone. If fewer than 10 communities were assigned to a given grid square, that grid square was merged with the neighboring square with the fewest number of communities (randomly breaking ties). We chose a finer resolution for countries with a larger number of deaths (4 × 4 for Tanzania, 4 × 5 for Malawi, and 5 × 5 for Niger), which resulted in 10 zones for each country (after merging as needed). In sensitivity analyses, we examined all combinations of 4, 5, and 6 latitude and longitude grid divisions for each country (729 possible grids in total), conducting four replications for each. The smaller TANA and PRET studies were not subdivided.

We tested the association between mortality reduction (comparing azithromycin and placebo communities) and baseline mortality as follows. For each zone, we estimated the log relative mortality rate and the average mortality rate in both azithromycin and placebo communities. Because some zones could contain zero deaths, we used the shrinkage estimator $\log\frac{\left(n_{1}+1/2\right)/T_{1}}{\left(n_{2}+1/2\right)/T_{2}}$, where *T*_*i*_ is the person-time at risk in treatment group *i* and *n*_*i*_ is the number of deaths in group *i*. Asymptotic errors of the mortality rate ratio were computed.^13^ We modeled the treatment effect by zone using linear mixed effects regression, with the average mortality rate per zone as a fixed effect regressor. We used country, and when necessary study, as a random effect, and weighted each observation by the inverse of the estimated variance of the mortality rate ratio. Analyses were conducted in R (v. 3.5 for Macintosh, R Foundation for Statistical Computing, Vienna, Austria).

## RESULTS

Data were obtained from TANA, PRET, and the three countries of the MORDOR study. From TANA, 48 communities were included, providing 9,754 person-years of monitoring of children from the age of 1–5 years (in contrast to the primary analysis, for ages 1–9 years). Between May 2006 and May 2007, 62 deaths were observed in this age group. From PRET, 48 communities were included, providing 11,872 person-years of monitoring for individuals aged 6 months through 4 years. A total of 404 deaths were observed between June 2010 and January 2013. From MORDOR, 1,512 communities were included in the analysis, 323,302 person-years were monitored, and 5,020 deaths were observed from December 2014 to July 2017 (Table 1). Azithromycin coverage exceeded 80% (Table 1). For the MORDOR communities of Niger, Malawi, and Tanzania, we analyzed 12, 11, and eight zones, respectively. The median number of deaths by zone was 327 (range 102–516) for Niger, 87 deaths (range 29–202) for Malawi, and 34.5 deaths (range 7–116) for Tanzania. The country-specific median total person-time per zone was 11,142 (range 3,271–24,440) person-years for Niger, 10,186 (range 4,765–18,141) person-years for Malawi, and 8,311 (range 999–17,711) person-years for Tanzania.

**Table 1:** TANA, PRET, and MORDOR data used in mortality analysis

Study	Location	Communities	Individuals (baseline)	Fraction younger than 1 year (%)	Reported coverage (%)
MORDOR	Niger	594	76,490	9.3	92–98
PRET	Niger	48	13,488	10.9	79–96
MORDOR	Tanzania	614	35,226	6.2	80–91
MORDOR	Malawi	304	78,920	6.7	88–95
TANA	Ethiopia	48	9,754	0	81–89

MORDOR = Macrolides Oraux pour Réduire les Décès avec un Oeil sur la Résistance; PRET = Rapid Elimination of Trachoma; TANA = Trachoma Amelioration in Northern Amhara. The number of randomization units is given in the Communities column. In the TANA study, individuals younger than 1 year were not treated. Full details are provided elsewhere.^1,2,4^

**Figure 1.**
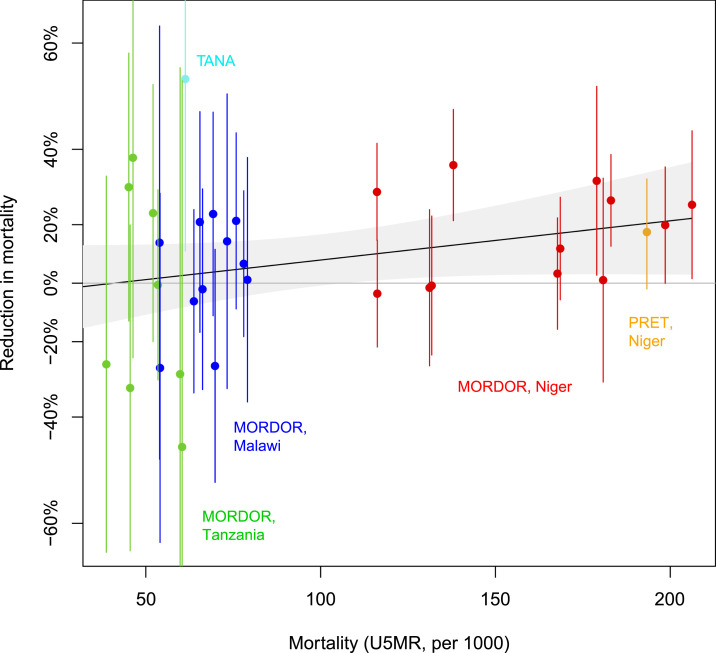
Azithromycin effect vs. mortality in three studies. The effect of azithromycin (vertical axis) is expressed as the natural logarithm of the relative rate (with larger values indicating larger benefit) in a geographic zone (see text for details). Mortality is depicted as the rate (azithromycin and placebo communities) in the given geographic zone, transformed to be comparable to an under-five mortality rate (per 1,000). Conversion to an approximate under-five mortality rate *u* was accomplished by *u* = *Yr* + *n*, where *Y* is the number of years of observation in each study, *r* is the estimated annual mortality rate in the given zone, and *n* is the reported neonatal mortality rate for the given nation (Ethiopia, Tanzania, Malawi, or Niger). Neonatal mortality rates from the World Bank were used in this calculation (https://data.worldbank.org/indicator/SP.DYN.IMRT.IN, accessed 22 December 2018). Statistical inference was not based on these neonatal mortality rates. Zone-specific neonatal mortality rates are not available. The black line and gray bands represent a descripive lowess smoothed and standard error.

Figure 1 shows the effect sizes as a function of baseline mortality. We found no convincing evidence that the baseline mortality rate modified the effect of azithromycin on mortality (*P* = 0.12). This finding was unchanged when controlling for the mean age of the enrolled children in each zone (*P* = 0.11). We also found modest evidence of a difference when we restricted to the three MORDOR sites (*P* = 0.039) combined (although not compelling, given multiple comparisons correction). We found no evidence of a difference when we restricted to Niger alone (*P* = 0.34). In an analysis restricted only to children younger than 1 year in MORDOR, there was no evidence of effect modification by the baseline mortality rate (*P* = 0.60). When restricting to children aged 2–4 years, we find *P* = 0.018.

Similar findings were obtained for other choices of geographic zones. Using a 4 × 4 grid for all countries yielded an overall nonsignificant *P*-value of 0.078, whereas a 5 × 5 grid yielded *P* = 0.16. Examining all 729 possible combinations of grid sizes of four, five, or six divisions per axis (longitude or latitude) for the three countries, only 24.7% of these analyses yielded significance at the 0.05 level.

## DISCUSSION

In this analysis, we found no compelling evidence that the underlying mortality rate modified the effect of azithromycin on child mortality, although a modest effect is not inconsistent with our findings. We note, however, that although the MORDOR trial was not powered for detection of effect modification, the findings we reported previously showed stronger evidence when restricted to MORDOR sites alone. The findings of the trial exhibited considerable apparent heterogeneity in efficacy by country,^4^ but in the reported prespecified secondary analysis, country did not modify the effect of treatment within MORDOR. An alternative analysis of the MORDOR results which included data from other sources found broadly similar findings.^14^ Our procedure is similar to that of the Bland–Altman analysis, based on modeling the difference in mortality as a function of the mean (by zone), athough modest bias may remain.

In a broad sense, areas with higher overall mortality may have a larger proportion because of infectious causes. We might expect no discernable benefit to mass azithromycin distribution in areas of the developed world where infectious disease causes a smaller portion of childhood deaths. Our findings cannot precisely estimate the threshold at which mortality benefits become negligible, and do not imply a lack of benefit for communities at the lower end of the range observed here.

Higher mortality areas clearly have the most to gain in terms of absolute number of deaths averted, even if the relative efficacy of azithromycin was similar across mortality rates. Our findings support prioritization of high childhood mortality regions, without precluding efforts in regions with relatively lower childhood mortality.
